# 96. Maternal Colonization, Perinatal Exposure, and Neonatal Acquisition of Resistant Enterobacterales

**DOI:** 10.1093/ofid/ofad500.012

**Published:** 2023-11-27

**Authors:** Leena B Mithal, Alima Sajwani, Abigail Aaron, Aspen N Kremer, Erica Hartmann, Jack Sumner, Andrew Watson, Emily Miller, Mehreen Arshad

**Affiliations:** Ann and Robert H. Lurie Children's Hospital of Chicago, Chicago, Illinois; Ann & Robert H. Lurie Children's Hospital, Chicago, Illinois; Lurie Children's/Northwestern, Chicago, Illinois; Ann & Robert H. Lurie Children's Hospital, Chicago, Illinois; Northwestern University, Evanston, Illinois; Northwestern University, Evanston, Illinois; Northwestern University, Evanston, Illinois; Brown University, Providence, Rhode Island; Northwestern University/Lurie Children's Hospital of Chicago, Chicago, IL

## Abstract

**Background:**

Extended Spectrum Beta-Lactamase producing Enterobacterales (ESBL-E) are globally prevalent. Pregnant people colonized with ESBL-E are at risk of perinatally transmitting to neonates, in whom infections with these strains are associated with higher mortality, morbidity and health care costs. In this study, we aimed to estimate the rate of gut colonization of AmpR-E (ampicillin resistant Enterobacterales) and ESBL-E in a population of healthy parent-infant dyads in the Chicago area and investigate the genetic characteristics of ESBL-E.

**Methods:**

Pregnant persons anticipating vaginal birth at two Chicago area hospitals were enrolled. Patients with preterm delivery (< 35 weeks), fever during labor, scheduled cesarean delivery, antibiotic use in the 3rd trimester, and infant NICU admission were excluded (Fig 1). Pregnancy and birth history were obtained. Parental vaginal and rectal swabs, and infant stool samples were collected and screened for ESBL-E by plating on MacConkey with ceftriaxone. Whole genome sequencing (WGS) and analysis was conducted on the ESBL-E isolates.

**Figure 1.**
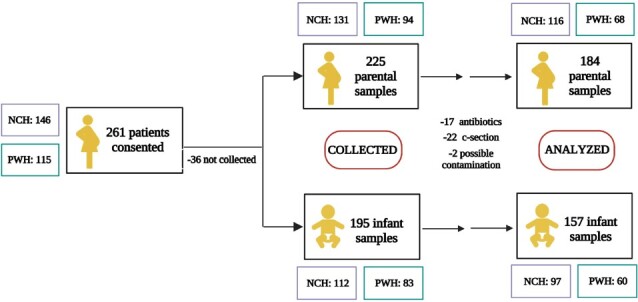
Patient cohort: enrollment and parental/infant samples.

Northwest Community Hospital (NCH), Northwestern Medicine Prentice Women’s Hospital (PWH). Final “n” for analysis was 184 parental and 157 infant samples. Infant samples were collected during hospital admission (1-2 days of life) and/or at 7-10 days of life.

**Results:**

Between July 2020 and April 2022, 261 parent-infant dyads were enrolled with 184 parental samples and 157 infant stool samples analyzed. Rate of parental AmpR-E gut colonization was 97% (179/184). Rate of parental and infant ESBL-E gut colonization was 14% (26/184) and 7% (11/157) respectively. Perinatal transmission of ESBL-E was 50% (11/22) (Fig 2). No clinical variables were significantly associated with parental ESBL-E colonization or perinatal transmission, although our power was limited (Table 1). Type of nutrition (exclusive formula feeding) was significantly associated with perinatal transmission of AmpR-E to infant (p=0.015). WGS of the ESBL-E showed that 19/42 isolates were *E. coli*, and transmission primarily occurred from parents colonized with ESBL *E. coli* (Fig 3).

**Figure 2.**
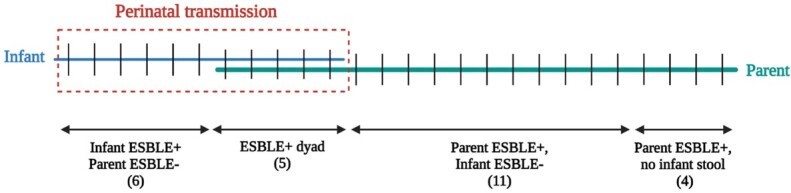
Mother and infant colonization with ESBL-E.

Red dotted line represents perinatal transmission of ESBL-E. If infant stool was positive for ESBL-E, perinatal transmission was assumed from ESBL-E positive mother despite maternal rectal swab negative for ESBL-E in culture (n=6).

**Table 1:**
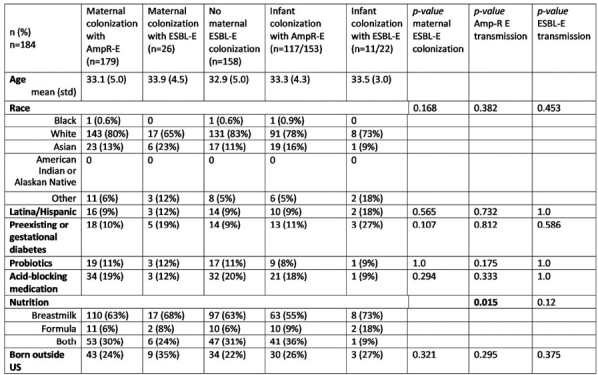
Characteristics of Mother-Infant Dyads by Colonization and Perinatal Transmission.

Comparisons were by Chi-square and Fisher’s Exact Tests, as appropriate. For purpose of data analysis, if infant stool was positive for ESBL-E, mother was considered ESBL-E positive even if rectal swab did not yield ESBL-E in culture.

**Figure 3.**
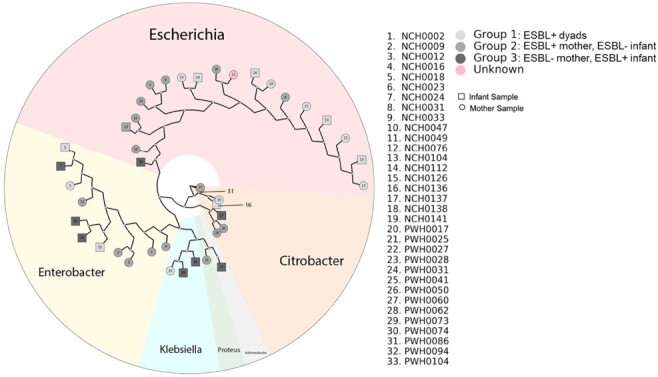
Whole genome sequencing of the ESBL Enterobacterales isolates.

Group number is indicated by grayscale color as indicated in legend. WGS showed that 45% were E. coli (19/42), followed by Enterobacter (11), Citrobacter (7), and Klebsiella (3).

**Conclusion:**

This study is the first of its kind in U.S. The burden of ESBL-E colonization in pregnant people is significantly higher than other developed countries (e.g., Norway, 2.9%) and comparable to countries with a high burden of ESBL-E (e.g., Sri Lanka, 17%) The impact of breastfeeding on infant colonization may be through mediation of microbiome but requires further study. ESBL *E. coli* are adept in perinatal transmission.

**Disclosures:**

**All Authors**: No reported disclosures

